# Transferring Sensor-Based Assessments to Clinical Practice: The Case of Muscle Synergies

**DOI:** 10.3390/s24123934

**Published:** 2024-06-18

**Authors:** Alessandro Scano, Valentina Lanzani, Cristina Brambilla, Andrea d’Avella

**Affiliations:** 1 Institute of Intelligent Industrial Systems and Technologies for Advanced Manufacturing (STIIMA), Italian Council of National Research (CNR), 20133 Milan, Italy; valentina.lanzani@stiima.cnr.it (V.L.); cristina.brambilla@stiima.cnr.it (C.B.); 2 Laboratory of Neuromotor Physiology, IRCCS Fondazione Santa Lucia, Via Ardeatina 306-354, 00179 Rome, Italy; a.davella@hsantalucia.it; 3 Department of Biology, University of Rome Tor Vergata, Via della Ricerca Scientifica, 00133 Rome, Italy

**Keywords:** muscle synergies, kinematic synergies, functional synergies, clinical assessment, instrumental assessments

## Abstract

Sensor-based assessments in medical practice and rehabilitation include the measurement of physiological signals such as EEG, EMG, ECG, heart rate, and NIRS, and the recording of movement kinematics and interaction forces. Such measurements are commonly employed in clinics with the aim of assessing patients’ pathologies, but so far some of them have found full exploitation mainly for research purposes. In fact, even though the data they allow to gather may shed light on physiopathology and mechanisms underlying motor recovery in rehabilitation, their practical use in the clinical environment is mainly devoted to research studies, with a very reduced impact on clinical practice. This is especially the case for muscle synergies, a well-known method for the evaluation of motor control in neuroscience based on multichannel EMG recordings. In this paper, considering neuromotor rehabilitation as one of the most important scenarios for exploiting novel methods to assess motor control, the main challenges and future perspectives for the standard clinical adoption of muscle synergy analysis are reported and critically discussed.

## 1. Introduction

Sensor-based assessments in medical practice and rehabilitation include the use of measurement of physiological signals such as electroencephalography (EEG), electromyography (EMG), electrocardiography (ECG), and near-infrared spectroscopy (NIRS), and the recording of movement kinematics and interaction forces [1]. Even though these measurements are commonly employed in clinics for assessing patients, some of them have not been fully exploited [2]. In fact, while instrumental assessments of patients may shed light on mechanisms underlying physiopathology and motor recovery, they have been used mostly in research studies, with a reduced impact on clinical practice, at least for some of these techniques [3]. This is especially the case for muscle synergies, a multichannel EMG-based analysis technique method for the evaluation of motor control commonly used in neuroscience. Muscle synergy refers to the coordinated activation of groups of muscles working together to produce specific movements or perform a particular task [4]. The concept of muscle synergies is based on the idea that the nervous system organizes and controls complex movements by recruiting and coordinating the activity of multiple muscles as functional units or modules [5]. In this way, rather than controlling each muscle involved in a movement individually, the nervous system simplifies the control process by organizing muscles into synergistic groups and flexibly combining their activation. These muscle synergies help streamline motor control and contribute to the efficiency of movements. Thus, muscle synergies are often studied in motor neuroscience to understand how the nervous system orchestrates movements. Researchers use muscle synergy-based approaches to analyze muscle activity and movement patterns during various tasks, finding applications in many clinical scenarios [6], including post-stroke patients [7,8,9], Parkinson’s disease [10], cerebral palsy [11], and others. Understanding muscle synergies may have implications for many fields such as rehabilitation [12], sports science [13], and robotics [14], as it provides insights into how the nervous system controls and coordinates movements [15]. Muscle synergy approaches have been used in many clinical applications. First, they have been used to investigate different tasks. In fact, muscle synergies have been identified as biomarkers of cortical damage in stroke patients during walking [16], showing that patients usually have a lower complexity in motor control. Other studies expanded synergistic assessment to upper limb movements, finding that shoulder muscle synergies are altered in hemiplegic patients [9,17]. Secondly, muscle synergies have been embedded into multidomain evaluations. In fact, muscle synergy assessment has been coupled with other signals, such as kinematics and EEG, to provide a comprehensive evaluation of patients [18,19]. Multiparameter approaches also include muscle synergies with spinal map activities, connecting EMG recorded at muscle level to the spatiotemporal motoneuronal activity in patients with spinal cord injuries [20]. Muscle synergies have also been employed to describe device-assisted rehabilitation. In fact, a field that has benefited from synergistic approaches is robotic rehabilitation, in which muscle synergies have been used to assess motor control improvement, showing that robotic treatment has beneficial effects on upper limbs [21,22] and hands [23]. Rehabilitation in virtual reality environments has also been assessed with muscle synergies in post-stroke patients [24] and children with cerebral palsy [25]. Lastly, muscle synergies have been used to guide functional electrical stimulation (FES) controller during lower limb rehabilitation [26] and to control myoelectric hand prosthesis [27].

However, despite their increasing diffusion in motor neuroscience research, the impact of synergies is still limited. Indeed, research findings and innovations reached in this field have had few effects on clinical scenarios or social and economic aspects of society, since the practical application of synergies is still very limited. From the analysis of the current literature, which is very wide and whose systematic report is beyond the scope of this contribution, fundamental issues emerge, showing that, although the method of muscle synergy analysis is spreading, it is far from being fully exploited in clinical scenarios. Some limitations are more evident, such as the limited number of participants in the studies; other limitations are subtler and include the use made of synergies merely as an evaluation metric. In addition, although some reviews that summarize the findings are available for many sub-fields of applications of muscle synergies, quantitative meta-analyses have not been provided. Therefore, with this contribution, we try to address the main issues that emerged from our screening by discussing several challenges that have only partially been met so far by the scientific and clinical communities.

In this study, referring mainly to neuromotor rehabilitation as the most promising scenario for applying synergistic approaches, the main challenges for the standard clinical adoption of muscle synergies are reported and critically discussed. Thus, the aim of the study is to summarize the main challenges needed to transfer muscle synergies for major exploitation in the clinical field.

## 2. Challenges

**Figure 1:**
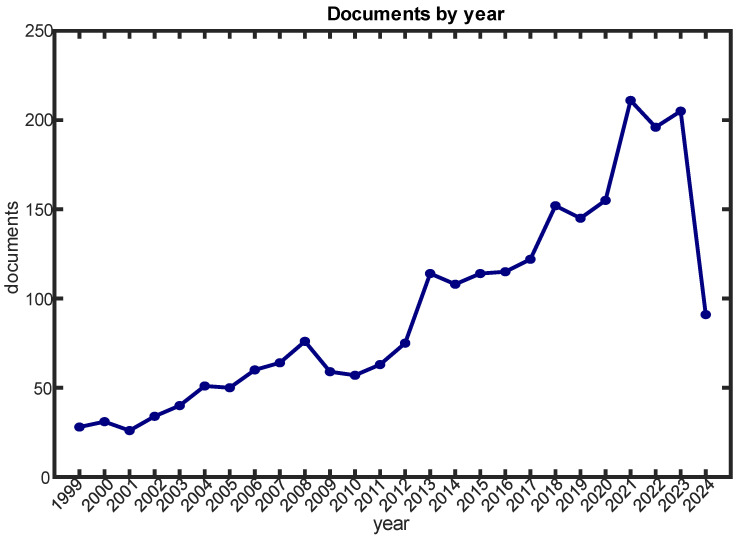
Number of papers found on Scopus when using “muscle AND synergy OR synergies” as keyword for the query and searching in abstract, keywords, and title, updated in April 2024.

The literature that is available regarding muscle synergies has been screened to identify the most representative work published so far about muscle synergies, especially regarding possible application to clinical scenarios. The corpus of available studies is very wide. Muscle synergies have become a major topic in neuroscience, as portrayed in Figure 1, which shows the number of English papers published in indexed journals from 1999 available in Scopus (updated until 30 April 2024). The subject area is limited to medicine, neuroscience, engineering, and computer science; the document type is limited to articles and reviews; and the language is limited to English.

The main added value of muscle synergies regards the comprehension and quantification of neuro-motor variables in the domain in which they are generated. In fact, standard clinical scales and kinematic analysis only investigate the output of the non-linear neuro–muscle–skeletal system dynamics, disregarding the neural variables and processes that account for such output. This is reflected in the consideration that most of the rehabilitative approaches focus on improving task performance and/or joint performance to indirectly improve neural activation, while a more direct approach should focus on assessing the patient’s neural commands and observe which kinematic coordination will result [7,28]. Whether muscle synergy analysis is more useful than conventional clinical metrics for assessing neurological patients’ severity remains unclear; however, further support for the need of muscle synergies comes from the motor abundance principle [29], which states that similar motor outputs are possibly generated by multiple manifolds of muscle activations [30]. In this way, the investigation of higher levels (with respect to kinematics) of the hierarchical and modular organization of the neuro-motor system is required to fully understand the system.

Surprisingly, despite their increasing diffusion in motor neuroscience research, the impact that synergies have reached (intended as the influence, significance, or effect of research findings) is still poor and is limited mainly to basic research. It follows that clinical, practical, real world, social, and economic impacts of the method are still very limited. Thus, a great challenge for the field is to transfer the synergistic approaches from basic research to clinical use, which could be one of the most valuable applications, if not the most attractive, of this method.

**Figure 2:**
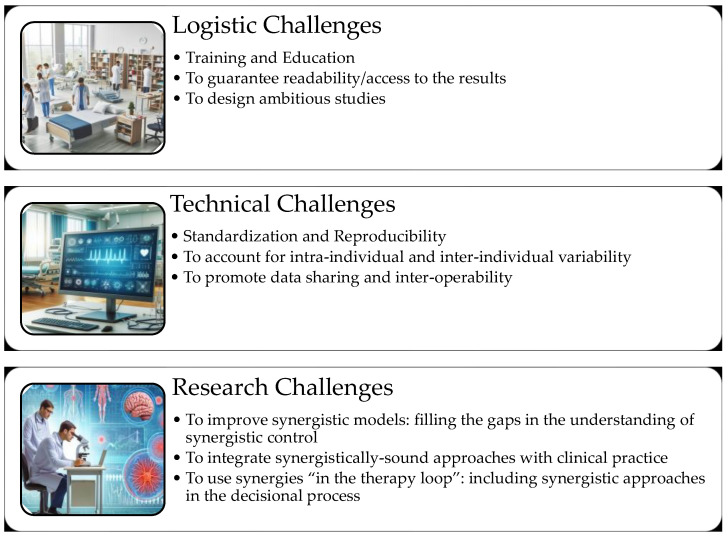
A summary of the challenges to be faced as summarized in this work. Logistic challenges include training and education, to design ambitious studies, to guarantee readability of the results. Technical challenges include to guarantee standardization and reproducibility, to account for intra-individual and inter-individual variability, to promote data sharing and inter-operability. Research challenges include to improve synergistic models, filling the gaps in the understanding of synergistic control, to integrate synergistically sound approaches with clinical practice, to use synergies “in the therapy loop”, including synergistic approaches in the decisional process.

The screening of the literature allows to identify three main groups of challenges connected to the adoption of muscle synergies in clinical scenarios: logistic challenges, technical challenges, and research challenges (Figure 2).

Logistic Challenges. They include the resources needed and organizational issues to be solved to make the method compatible with clinical scenarios. The following logistic challenges have been identified:

- Training and education;
- Design of ambitious studies;
- Guarantee of readability/access to the results.

Technical Challenges. They include the technical application of the method and the scientific activities connected to the method. The following technical challenges have been identified:

- To guarantee standardization and reproducibility;
- To account for intra-individual and inter-individual variability;
- To promote data sharing and inter-operability.

Research Challenges. They include wider scoped questions that relate to the improvement and the full understanding of the method itself and its application to clinical scenarios. The following research challenges have been identified:

- To improve synergistic models, filling the gaps in the understanding of synergistic control;
- To integrate synergistically sound approaches with clinical practice;
- To use synergies “in the therapy loop”, including synergistic approaches in the decisional process.

### 2.1. Logistic Challenges

#### 2.1.1. Training and Education

Synergy analysis is a highly multidisciplinary approach to understanding neuro-motor control, which requires knowledge in many fields. A general rule for success in multidisciplinary fields is to gather multidisciplinary scientific teams able to cover all the areas of knowledge needed to gather data, perform the analysis, and interpret the results [31]. To correctly acquire multichannel EMG signals, the first step is to guarantee accurate synergistic assessment. It has been shown that specific professional figures might be instructed to perform signal recordings rather than relying only on bioengineers or on medical personnel without a specific training [32,33]. Furthermore, there is a need for bioengineers who are instructed and updated on the variety of steps for synergy extraction and have a wide view of the available literature, including signal processing, algorithms for extraction, and synergistic models. Experienced physicians are also needed to drive scientific questions and to support the selection of the best pipelines and algorithms to adopt for synergy analysis, as they encompass a physiological interpretation of the findings [34]. It is of paramount importance that all these figures work as one to improve the impact of synergy-based studies, while much work in the field is carried on by a subset of them.

#### 2.1.2. To Design Ambitious Studies

In terms of statistical significance, most of the synergy-based studies cannot compare with standard clinical studies that use clinical scales for patients’ assessments, e.g., to evaluate the outcome of standard occupational therapy or the effect of drugs. This is probably due to their intrinsic design based on the nature of research, highly relying on pilot and concept work. Indeed, a recent review highlighted that clinical studies with muscle synergies involve only about 15 patients on average [6]. Other factors may include the relatively long time needed for preparing the set-up and instrumenting patients with sensors, and the complexity and effort needed for analyzing the data. A specific field that well exemplifies this limitation is robotic rehabilitation, where, according to the authors’ best knowledge, the study based on synergistic evaluation with the largest number of enrolled patients is from Lencioni et al. [21], originally featuring 40 post-stroke patients (32 of whom completed the protocol and were analyzed). All the other studies on robotic rehabilitation enrolled a lower number of patients, thus limiting the significance of the results, even when they were based on very refined designs and algorithms. Consequently, systematic reviews conducted on muscle synergy studies are usually based on proof of concept studies rather than on structured clinical trials.

Moreover, in many clinical contexts, multichannel EMG (eight or more channels) might not be available, since this kind of equipment can be very expensive for clinics [35]. It is indeed necessary that many clinical structures decide to equip themselves with such instruments, with the consideration of muscle synergies as a cost-effective technique to be employed and supported.

#### 2.1.3. To Guarantee Readability/Access to the Results

Synergistic approaches generate output data that are hard for engineers to read and interpret, as they lack neurological or physiology knowledge, and for physicians, as they are not always fully aware of the details of each synergy extraction method and how it impacts the interpretation of the outputs. For example, the level of impairment and the side of lesion in stroke patients may influence the synergy structure [36,37]; therefore, understanding of the pathological state is needed for a correct interpretation of synergy analysis. However, in a few works, the data regarding the type of lesion are available. A fundamental tool to fill this gap may come from the adoption of detailed reports that summarize the main findings achieved in the study. Synthetic reports should be comprehensive of explanations and physiological interpretation of the modifications of synergies induced by treatments or when comparing conditions, coupling clinical and synergistic findings. Such reports might also involve synthesis of the outputs at different time frames, for example, in longitudinal studies. They might also exploit artificial intelligence or reasoning systems to provide accurate reports, shared across studies if based on the same evaluation systems.

### 2.2. Technical Challenges

#### 2.2.1. Standardization and Reproducibility

One of the most crucial challenges regards the establishment of standardized methods and pipelines to extract synergies from EMG signals. This process is made of many steps and includes electrode positioning, signal filtering (high-pass and low-pass cut-off frequencies, whose effects have been studied systematically, e.g., in [38]), EMG normalization (according to various techniques), data pooling (i.e., whether data should be averaged, concatenated, or analyzed separately [39]), and other preprocessing steps. Two further analysis steps are particularly crucial: the choice of the extraction algorithm, which may vary depending on the aim and on the study design, and the choice of the criteria for selecting the number of synergies. Both these design selections intrinsically impact in a fundamental way the interpretation of the data and the clinical application of synergistic models. There are many algorithms that can be used, such as the synchronous or spatial model [40,41], the temporal model [42,43], the spatiotemporal model [44], the space-by-time model [45], and the autoencoder [46]. Interestingly, each model may capture features that help describe synergistic control under specific assumptions, and thus intrinsically incorporate a specific knowledge and understanding of the physiology underlying neural control of movement. A clear example of this can be found in Berger et al. [47,48], who showed that the spatial model could not identify specific impairments in the motor organization of patients with cerebellar damage. It was instead shown that temporal and spatiotemporal synergies could capture relevant modifications. Despite the variety of algorithms and approaches available, almost no other study has compared synergy extraction approaches to understand the range of applicability and efficacy of each algorithm on clinical data, limiting the analysis to one synergistic model per work. Similarly, no guideline is available regarding which models should be coupled to specific conditions or pathologies. It follows that one major challenge would be to couple the most appropriate algorithm(s) for the investigated domain.

Another still unresolved challenge is the selection of the optimal number of synergies. Even though a large variety of methods have been proposed and tested, including standard VAF/R^2^ thresholds, minimum VAF variation, a priori selection, linear fit, multi-VAF resolution analysis [49], and others, it is still a matter of debate whether an optimal number of synergies can really be defined, and how [50]. Indeed, since synergies are expected to be organized at the neural level, one would expect that it would be possible to define univocally the “right” number of synergies, even though recent work suggests that a multiresolution analysis might be more suitable and accurate for interpreting synergy data [51]. How these problems relate to observed findings such as synergy merging and fractionation [9] is also a matter of investigation for a better understanding of motor control to foster the clinical application of synergistic approaches.

Some attempts to improve reproducibility and standardization have been made by proposing reference evaluation protocols [51] or by distributing toolboxes for synergistic control in some applications, including upper limb [52], general purpose and lower limb [53], hand [54], and exoskeleton walking [55]. However, these attempts of standardizing synergy analysis are mostly from a single group or a few research groups rather than from a wide consortium of synergy users.

#### 2.2.2. To Account for Intra-Individual and Inter-Individual Variability

Movement shows intrinsic variability that can be observed both at the intra-individual level, due to the differences in the multiple executions of the same task by the same subject [56], and at the inter-individual level, related to the variation of motor patterns when multiple subjects perform the same task [57]. Movement variability is reflected in synergy variability [58]. A very important need is to build a definitive highly reliable quantification of the intra-individual and inter-individual variability of synergies in order to define normative datasets that allow to evaluate, in a comparative sense, the results with referable data. Generating such datasets would implicitly constrain many protocol choices to a standard procedure (e.g., number and positioning of the EMG electrodes) and allow to quantify at what extent differences and modifications are due to inherent modifications of the neural system and which are due to intrinsic physiological variability between trials and subjects. Movement variability may affect the correct interpretation of synergies, capturing changes that are not related to neurological improvements [59]. This limitation afflicts many longitudinal studies and has rarely been quantified in previous works, even though it is a fundamental step toward an aware use of the method.

#### 2.2.3. To Promote Data Sharing and Inter-Operability

As clinical scales show some limitations such as poor resolution in evaluating motor capabilities and potential inter-operator biases [60], they are in general completely inter-operable between clinical centers and standardized in their formulation. The same standardization is not found with muscle synergies [61]. Thus, transferring synergies to clinical practice would require individuating common and shared platforms for data sharing; individuating common and shared data formats; and building publicly interconnected repositories. All these aspects are at the moment completely missing and contribute to making the results of many studies difficult to compare. Lastly, there is also a lack of shared databases that can be exploited by the scientific community; in fact, the vast majority of the data underlying synergistic studies are not published in shared repositories (probably also due to the limited number of subjects available).

### 2.3. Research Challenges

#### 2.3.1. To Improve Synergistic Models, Filling the Gaps in the Understanding of Synergistic Control

Improving synergistic models would facilitate clinical practice as it would simplify the choice of methods and algorithms to be employed to study each clinical condition. Several communications and reviews have pointed out specific directions to follow, which are summarized below.

**The neural basis of synergistic control**. More work should be carried out to understand whether synergistic control reflect neural implementation and what are the neural bases underlying it [62]. Much effort has been made to understand the roles of the spinal cord, brain stem, and motor cortical areas in the activation, organization, and fine-tuning of muscle synergies. A recent review suggested to constrain models with neurophysiological knowledge [63]. Discerning the applicability of spatial, temporal, and spatiotemporal models should also be further considered. Moreover, the alteration in muscle synergies might be dependent on the site of lesions, the severity of impairment, and the stage of disease [64], and these data are often not considered in the available studies.

**Synergy models**. It has also been suggested to encourage the concurrent use of various synergy models–spatial, temporal, and spatiotemporal synergies. The number of synergies represents either the dimension of the spatial structure or the number of independent temporal patterns, and it has been observed that these two aspects are often mixed in the analysis. To select a number, criteria based on noise estimates through bootstrap methods, reliability of analysis results, or functional outcomes of the synergies provide interesting substitutes to criteria solely based on variance thresholds [65].

**Link to the task space**. A major step toward the conception of synergy-driven approaches may also come from the linking of neural synergies to the task space variables [14], which has been addressed with recent novel algorithms that extend the concept of synergies by incorporating task variables to the neural drive [66,67,68] or factorizations based on multidomain approaches [18]. In such approaches, muscle weights are flanked with task weights (typically kinematic or force) that show how the neural synergy results in a task output.

**The role of non-linearities**. Most of the algorithms used so far naturally employ a linear combination of synergies to produce purposeful movement. While this is compatible with most of the observations made on humans and on animals, it is possible that future work will have to consider more complex models, and thus they may require to adopt non-linear models, such as the recently used autoencoder-based approaches [46], kinematic–muscular synergies [66], or information and network theory to measure the task relevance of the synergies [67].

#### 2.3.2. To Integrate Synergistically Sound Approaches with Clinical Practice

One complex challenge to face is to find a proper trade-off between standard clinical assessments and synergistic approaches by harmoniously integrating the two frameworks. In fact, finding a common ground between structured protocols typical of research laboratories and clinical demands poses a challenge that has not yet been solved. In laboratory studies, volunteers are typically requested to follow structured protocols (e.g., standard multitarget reaching movements exploring many directionalities throughout many repetitions [69] or, in any case, a wide variety of movements and task repetitions). In a clinical environment, a sufficient level of motion variability should be persevered (i.e., when possible, a reasonable number of tasks and repetitions should be performed to guarantee that synergistic extraction is adequately representing the repertoire of modules available to subjects) keeping in mind the limitations due to patients’ motor capability and cooperation [6]. A reasonable trade-off might be an approach combining synergies with functional movements resembling clinical scales [70,71], even though more should be carried out to find a consensus.

#### 2.3.3. To Use Synergies “in the Therapy Loop”, including Synergistic Approaches in the Decisional Process

So far, synergies have been used only as an evaluation tool in the vast majority of studies [7]. Typically, synergies are used as a mere evaluation method to compare two conditions (e.g., pre- or post-therapy synergies in longitudinal studies; robot-assisted synergies, and free movement in single-session studies). However, even though such approaches are valuable in principle, they do not fully exploit the potential of a synergistic approach, as they evaluate synergies as an output of an intervention conceived in another domain. In fact, there is a lack of synergy-based rehabilitation protocols, i.e., protocols conceived to promote, restore, or reshape specific synergies or synergy combinations. So, the neural frame is used only to evaluate the effects of therapies and interventions that are not conceived in the same domain but rather in the tridimensional space or in the joint domain inspired to human kinematics. Few studies have focused on developing effective ways to use muscle synergies as a training target or a control input signal for intervention systems; however, changes in muscle synergies would maximize the motor function improvement [7]. Creating synergistic-based rehabilitation protocols might be an example on how to foster such a view, for example, by changing the destination and use of the robot due to synergistic assessment, to customize therapies on the basis of synergy clustering in order to restore specific synergies, or to reshape temporal commands in case synergies cannot be changed (as in chronic patients). Another option would be, in the robotic field, to create control-based algorithms based on synergistic approaches [72] or to tune therapies or assistance depending on the recorded synergies.

A summary of the main needs and lessons learned to transfer muscle synergy to a standard suitable for clinical practice are shown in Table 1.

## 3. Perspectives

### 3.1. Logistic Challenges

Logistic challenges that have to be addressed include the need for professional resources to make the method compatible with clinical scenarios. A multidisciplinary team is necessary to apply synergy analysis and to understand the neuro-motor control. The knowledge needed for data gathering, performing the analysis, and interpreting the results should be covered by qualified experts in the field. Therefore, expert professionals are needed to correctly acquire multichannel EMG signals, as a fundamental preliminary step for the data analyses aiming at synergy extraction in order to reduce signal noise, cross-talk, artefacts, and inaccuracies. Then, the analyses should be supported by bioengineers who are updated on synergistic models and data processing and able to select the pipelines that best fit the aims of the work. An appropriate physiological interpretation and a selection of relevant clinical implications should instead be led by physicians, providing insights from a medical point of view. Only a constant interaction with all these professional figures may guarantee the exploitation of the full potential of the method. From this perspective, readability of the results of synergistic analysis becomes a crucial issue. Indeed, the outputs of the analysis can be non-trivial to read and interpret for both engineers and physicians. Therefore, the main findings should be summarized in detailed reports that provide comprehensive explanations and physiological interpretation of the modifications of synergies induced by treatments or when comparing conditions, adding clinical interpretation to synergistic outputs according to systematic criteria.

Moreover, comprehensive studies applying muscle synergy analysis are not available in the literature and are usually limited to pilot research studies. Therefore, more structured studies in which the longitudinal course of therapy is investigated should be designed and a large number of participants and patients should be enrolled so that the significance and generalizability power of the studies increase. A last barrier to overcome refers to the non- negligible cost of the equipment. For a systematic adoption of muscle synergy analysis, clinical structures need to equip themselves with such instruments and consider muscle synergies as a cost-effective technique to be employed and supported.

### 3.2. Technical Challenges

The standardization and the reproducibility of the methodological approach is fundamental for increasing the applicability of synergy analysis in clinical scenarios. To overcome this challenge, it is necessary to generate consensus guidelines for processing and protocols, including the pre-processing steps, the choice of the synergy model and the corresponding factorization algorithm, the criteria for selecting the number of synergies, and others. These guidelines may be associated with specific research questions in which different methodological steps are needed, so that guidelines are specified for each investigation. Defining technical standards and reducing custom choices will help to spread the use of these techniques, and especially increase the possibility of comparisons between studies. The adoption of freely usable and distributed synergy-based toolboxes [52] should be encouraged to guarantee easy-to-use methods for all researchers and clinicians and promote common background between researchers.

Another important technical aspect poorly assessed in the literature is the need to quantify the intra-individual and inter-individual variability of synergies, to avoid the misinterpretation of synergistic output by assuming that changes or modifications to synergies are due to neurological improvements rather than physiological differences between experimental sessions or experimental biases (e.g., imperfect inter-session electrode positioning). Therefore, normative datasets need to be created to investigate intra-session, inter-session, intra-subject, and inter-subject variability and publicly shared in order to build normative databases of muscle synergies that can be used as reference. The limited data sharing and inter-operability are also technical challenges to face, that add further need of standardization to the previously described limitations. To promote data sharing, it is fundamental to identify common data formats that can be used by anyone and to identify common platforms in which data can be shared. Publicly interconnected repositories can be created for this purpose; indeed, this will help to exploit research findings and to compare studies.

### 3.3. Research Challenges

From a research point of view, synergistic models need to be improved to facilitate their use in clinical practice. First of all, the role of neural structures, such as the spinal cord, the brain stem, and motor cortical areas, in implementing synergistic control should be better understood so that synergy models can be linked more directly to neuromotor physiology. Moreover, since different synergy models provide different insights into motor control, multiple synergy models should be implemented concurrently to provide comprehensive perspectives when describing motor control in a specific scenario. Another important step to improve the interpretation of the outcomes of synergy models is the inclusion in the model of a direct link to the task space, showing how neural synergies are mapped into task outputs. Some models have been developed [66], but more research needs to be performed in future to overcome limitations such as linearity of the models.

One complex challenge to face is to integrate synergistically sound approaches with clinical practice. Indeed, a trade-off between standard clinical assessments and synergistic approaches should be found. In fact, a sufficient variety and quantity of movements is necessary for extracting reliable synergies; however, this need should consider the limitations due to patients’ motor capability and cooperation. Therefore, consensus protocols should be generated to harmonize the requirements of clinical treatments with the requirements of synergistic approaches. One possible option is to use assessments based on functional movements, resembling those performed in clinical scales [71], that are already administered to patients when they are evaluated in clinics with standard protocols.

Finally, the potential of the synergistic approach has not been fully exploited, as synergies have been used only as an assessment method. Indeed, the synergistic approach should be included in the decisional process performed by clinicians in selecting specific rehabilitative programs. Synergy-based rehabilitation protocols, conceived to promote, restore, or reshape synergies, should be encouraged and their efficacy assessed. For example, the destination and use of a robotic operator for rehabilitation may be decided on the basis of synergistic assessment and control-based algorithms based on synergistic approaches. In this way, the effects of therapies and interventions are evaluated in the muscle synergy domain, with the aim of maximizing motor function improvement.

## 4. Conclusions

Many challenges arise when aiming at the systematic application of muscle synergies to clinical practice. However, to date, the challenges and issues presented in this work have not been addressed and solved all together, leaving a wide margin for improving the adoption of the method in clinical practice. There is a need for comprehensive and ambitious future studies addressing and solving these challenges. In fact, some of the challenges have been partially addressed but never with a comprehensive uniform vision, which is ultimately needed for transferring muscle synergy assessment to standard clinical practice.

## Figures and Tables

**Table 1:** Needs and lessons learned regarding the application of synergistic approaches in clinical environments.

Type of Challenge	Needs	Lessons Learned/Possible Solutions
Logistic	Training and Education	To correctly acquire multichannel EMG signals with personnel devoted to data acquisition. To enroll bioengineers to support analysis. They should be updated on synergistic models and pipelines. Physicians should drive clinical questions and needs.
Logistic	To design ambitious studies	To increase the number of structured studies. To enroll a large number of patients/healthy controls to increase study significance. To consider expensive equipment as cost-effective.
Logistic	To guarantee readability of the results	To provide synthetic reports for clinicians, including physiological explanations, multisession comparisons, etc., readable by technical and medical personnel.
Technical	To guarantee standardization and reproducibility	To generate consensus guidelines for processing and protocols. To provide freely usable synergy-based toolboxes. To limit custom choices in research by defining technical standards. To associate algorithms and pipelines to specific research questions.
Technical	To account for intra-individual and inter-individual variability	To build shared and public reference databases. To investigate intra-session, inter-session, intra-subject, and inter-subject variability.
Technical	To promote data sharing and inter-operability	To individuate common platforms for data sharing. To individuate common and shared data formats. To create publicly interconnected repositories.
Research	To improve synergistic models, filling the gaps in the understanding of synergistic control	To improve the understanding of the neural basis of synergistic control. To concurrently implement multiple synergy models. To create a link to the task space. To evaluate the role of non-linearities.
Research	To integrate synergistically sound approaches with clinical practice	To generate consensus protocols, at least for some classes of experiments. To harmonize the requirements of the clinics with the requirements of synergistic approaches.
Research	To use synergies “in the therapy loop”, including synergistic approaches in the decisional process	To create synergy-based rehabilitation protocols. To change the destination and use of the therapy/robot/treatment due to synergistic assessments. To create control-based algorithms based on synergistic approaches.

## Data Availability

Data are contained within the article.

## References

[1] Scano A., Guanziroli E., Brambilla C., Amendola C., Pirovano I., Gasperini G., Molteni F., Spinelli L., Molinari Tosatti L., Rizzo G. (2023). A Narrative Review on Multi-Domain Instrumental Approaches to Evaluate Neuromotor Function in Rehabilitation. Healthcare.

[2] Maura R.M., Rueda Parra S., Stevens R.E., Weeks D.L., Wolbrecht E.T., Perry J.C. (2023). Literature Review of Stroke Assessment for Upper-Extremity Physical Function via EEG, EMG, Kinematic, and Kinetic Measurements and Their Reliability. J. Neuroeng. Rehabil..

[3] Campanini I., Disselhorst-Klug C., Rymer W.Z., Merletti R. (2020). Surface EMG in Clinical Assessment and Neurorehabilitation: Barriers Limiting Its Use. Front. Neurol..

[4] Tresch M.C., Jarc A. (2009). The Case for and against Muscle Synergies. Curr. Opin. Neurobiol..

[5] Bizzi E., Cheung V.C.K., d’Avella A., Saltiel P., Tresch M. (2008). Combining Modules for Movement. Brain Res. Rev..

[6] Zhao K., Zhang Z., Wen H., Liu B., Li J., d’Avella A., Scano A. (2023). Muscle Synergies for Evaluating Upper Limb in Clinical Applications: A Systematic Review. Heliyon.

[7] Hong Y.N.G., Ballekere A.N., Fregly B.J., Roh J. (2021). Are Muscle Synergies Useful for Stroke Rehabilitation?. Curr. Opin. Biomed. Eng..

[8] Cheung V.C.K., Piron L., Agostini M., Silvoni S., Turolla A., Bizzi E. (2009). Stability of Muscle Synergies for Voluntary Actions after Cortical Stroke in Humans. Proc. Natl. Acad. Sci. USA.

[9] Cheung V.C.K., Turolla A., Agostini M., Silvoni S., Bennis C., Kasi P., Paganoni S., Bonato P., Bizzi E. (2012). Muscle Synergy Patterns as Physiological Markers of Motor Cortical Damage. Proc. Natl. Acad. Sci. USA.

[10] Mileti I., Zampogna A., Santuz A., Asci F., Del Prete Z., Arampatzis A., Palermo E., Suppa A. (2020). Muscle Synergies in Parkinson’s Disease. Sensors.

[11] Steele K.M., Rozumalski A., Schwartz M.H. (2015). Muscle Synergies and Complexity of Neuromuscular Control during Gait in Cerebral Palsy. Dev. Med. Child Neurol..

[12] Dipietro L., Krebs H.I., Fasoli S.E., Volpe B.T., Stein J., Bever C., Hogan N. (2007). Changing Motor Synergies in Chronic Stroke. J. Neurophysiol..

[13] Frère J., Hug F. (2012). Between-Subject Variability of Muscle Synergies during a Complex Motor Skill. Front. Comput. Neurosci..

[14] Alessandro C., Delis I., Nori F., Panzeri S., Berret B. (2013). Muscle Synergies in Neuroscience and Robotics: From Input-Space to Task-Space Perspectives. Front. Comput. Neurosci..

[15] Taborri J., Agostini V., Artemiadis P.K., Ghislieri M., Jacobs D.A., Roh J., Rossi S. (2018). Feasibility of Muscle Synergy Outcomes in Clinics, Robotics, and Sports: A Systematic Review. Appl. Bionics. Biomech..

[16] Clark D.J., Ting L.H., Zajac F.E., Neptune R.R., Kautz S.A. (2010). Merging of Healthy Motor Modules Predicts Reduced Locomotor Performance and Muscle Coordination Complexity Post-Stroke. J. Neurophysiol..

[17] Roh J., Rymer W.Z., Perreault E.J., Yoo S.B., Beer R.F. (2013). Alterations in Upper Limb Muscle Synergy Structure in Chronic Stroke Survivors. J. Neurophysiol..

[18] Pierella C., Pirondini E., Kinany N., Coscia M., Giang C., Miehlbradt J., Magnin C., Nicolo P., Dalise S., Sgherri G. (2020). A Multimodal Approach to Capture Post-Stroke Temporal Dynamics of Recovery. J. Neural. Eng..

[19] Belfatto A., Scano A., Chiavenna A., Mastropietro A., Mrakic-Sposta S., Pittaccio S., Molinari Tosatti L., Molteni F., Rizzo G. (2018). A Multiparameter Approach to Evaluate Post-Stroke Patients: An Application on Robotic Rehabilitation. Appl. Sci..

[20] Bellitto A., De Luca A., Gamba S., Losio L., Massone A., Casadio M., Pierella C. (2023). Clinical, Kinematic and Muscle Assessment of Bilateral Coordinated Upper-Limb Movements Following Cervical Spinal Cord Injury. IEEE Trans. Neural. Syst. Rehabil. Eng..

[21] Lencioni T., Fornia L., Bowman T., Marzegan A., Caronni A., Turolla A., Jonsdottir J., Carpinella I., Ferrarin M. (2021). A Randomized Controlled Trial on the Effects Induced by Robot-Assisted and Usual-Care Rehabilitation on Upper Limb Muscle Synergies in Post-Stroke Subjects. Sci. Rep..

[22] Tropea P., Monaco V., Coscia M., Posteraro F., Micera S. (2013). Effects of Early and Intensive Neuro-Rehabilitative Treatment on Muscle Synergies in Acute Post-Stroke Patients: A Pilot Study. J. Neuroeng. Rehabil..

[23] Scotto di Luzio F., Cordella F., Bravi M., Santacaterina F., Bressi F., Sterzi S., Zollo L. (2022). Modification of Hand Muscular Synergies in Stroke Patients after Robot-Aided Rehabilitation. Appl. Sci..

[24] Maistrello L., Rimini D., Cheung V.C.K., Pregnolato G., Turolla A. (2021). Muscle Synergies and Clinical Outcome Measures Describe Different Factors of Upper Limb Motor Function in Stroke Survivors Undergoing Rehabilitation in a Virtual Reality Environment. Sensors.

[25] Booth A.T.C., van der Krogt M.M., Harlaar J., Dominici N., Buizer A.I. (2019). Muscle Synergies in Response to Biofeedback-Driven Gait Adaptations in Children With Cerebral Palsy. Front. Physiol..

[26] Ferrante S., Chia Bejarano N., Ambrosini E., Nardone A., Turcato A.M., Monticone M., Ferrigno G., Pedrocchi A. (2016). A Personalized Multi-Channel FES Controller Based on Muscle Synergies to Support Gait Rehabilitation after Stroke. Front. Neurosci..

[27] Furui A., Eto S., Nakagaki K., Shimada K., Nakamura G., Masuda A., Chin T., Tsuji T. (2019). A Myoelectric Prosthetic Hand with Muscle Synergy-Based Motion Determination and Impedance Model-Based Biomimetic Control. Sci. Robot..

[28] Patel V., Craig J., Schumacher M., Burns M.K., Florescu I., Vinjamuri R. (2017). Synergy Repetition Training versus Task Repetition Training in Acquiring New Skill. Front. Bioeng. Biotechnol..

[29] Latash M.L. (2012). The Bliss (Not the Problem) of Motor Abundance (Not Redundancy). Exp. Brain Res..

[30] Kang N., Shinohara M., Zatsiorsky V.M., Latash M.L. (2004). Learning Multi-Finger Synergies: An Uncontrolled Manifold Analysis. Exp. Brain Res..

[31] Manca A., Cereatti A., Bar-On L., Botter A., Della Croce U., Knaflitz M., Maffiuletti N.A., Mazzoli D., Merlo A., Roatta S. (2020). A Survey on the Use and Barriers of Surface Electromyography in Neurorehabilitation. Front. Neurol..

[32] Merletti R., Temporiti F., Gatti R., Gupta S., Sandrini G., Serrao M. (2023). Translation of Surface Electromyography to Clinical and Motor Rehabilitation Applications: The Need for New Clinical Figures. Transl. Neurosci..

[33] Merletti R. (2024). Metrology in sEMG and Movement Analysis: The Need for Training New Figures in Clinical Rehabilitation. Front. Rehabil. Sci..

[34] McManus L., De Vito G., Lowery M.M. (2020). Analysis and Biophysics of Surface EMG for Physiotherapists and Kinesiologists: Toward a Common Language With Rehabilitation Engineers. Front. Neurol..

[35] Feldner H.A., Howell D., Kelly V.E., McCoy S.W., Steele K.M. (2019). “Look, Your Muscles Are Firing!”: A Qualitative Study of Clinician Perspectives on the Use of Surface Electromyography in Neurorehabilitation. Arch. Phys. Med. Rehabil..

[36] Pellegrino L., Coscia M., Pierella C., Giannoni P., Cherif A., Mugnosso M., Marinelli L., Casadio M. (2021). Effects of Hemispheric Stroke Localization on the Reorganization of Arm Movements within Different Mechanical Environments. Life.

[37] Roh J., Rymer W.Z., Beer R.F. (2015). Evidence for Altered Upper Extremity Muscle Synergies in Chronic Stroke Survivors with Mild and Moderate Impairment. Front. Hum. Neurosci..

[38] Kieliba P., Tropea P., Pirondini E., Coscia M., Micera S., Artoni F. (2018). How Are Muscle Synergies Affected by Electromyography Pre-Processing?. IEEE Trans. Neural Syst. Rehabil. Eng..

[39] Oliveira A.S., Gizzi L., Farina D., Kersting U.G. (2014). Motor Modules of Human Locomotion: Influence of EMG Averaging, Concatenation, and Number of Step Cycles. Front. Hum. Neurosci..

[40] Tresch M.C., Saltiel P., Bizzi E. (1999). The Construction of Movement by the Spinal Cord. Nat. Neurosci..

[41] Ting L.H., Macpherson J.M. (2005). A Limited Set of Muscle Synergies for Force Control during a Postural Task. J. Neurophysiol..

[42] Ivanenko Y.P., Poppele R.E., Lacquaniti F. (2004). Five Basic Muscle Activation Patterns Account for Muscle Activity during Human Locomotion. J. Physiol..

[43] Ivanenko Y.P., Cappellini G., Dominici N., Poppele R.E., Lacquaniti F. (2005). Coordination of Locomotion with Voluntary Movements in Humans. J. Neurosci..

[44] d’Avella A., Saltiel P., Bizzi E. (2003). Combinations of Muscle Synergies in the Construction of a Natural Motor Behavior. Nat. Neurosci..

[45] Delis I., Panzeri S., Pozzo T., Berret B. (2014). A Unifying Model of Concurrent Spatial and Temporal Modularity in Muscle Activity. J. Neurophysiol..

[46] Buongiorno D., Cascarano G.D., Camardella C., De Feudis I., Frisoli A., Bevilacqua V. (2020). Task-Oriented Muscle Synergy Extraction Using An Autoencoder-Based Neural Model. Information.

[47] Berger D.J., Masciullo M., Molinari M., Lacquaniti F., d’Avella A. (2020). Does the Cerebellum Shape the Spatiotemporal Organization of Muscle Patterns? Insights from Subjects with Cerebellar Ataxias. J. Neurophysiol..

[48] Berger D.J., Ferrari F., Esposito A., Masciullo M., Molinari M., Lacquaniti F., d’Avella A., Ibáñez J., González-Vargas J., Azorín J.M., Akay M., Pons J.L. (2017). Changes in Muscle Synergy Organization after Neurological Lesions. Converging Clinical and Engineering Research on Neurorehabilitation II.

[49] Pale U., Atzori M., Müller H., Scano A. (2020). Variability of Muscle Synergies in Hand Grasps: Analysis of Intra- and Inter-Session Data. Sensors.

[50] Ranaldi S., De Marchis C., Severini G., Conforto S. (2021). An Objective, Information-Based Approach for Selecting the Number of Muscle Synergies to Be Extracted via Non-Negative Matrix Factorization. IEEE Trans. Neural Syst. Rehabil. Eng..

[51] Longatelli V., Torricelli D., Tornero J., Pedrocchi A., Molteni F., Pons J.L., Gandolla M. (2022). A Unified Scheme for the Benchmarking of Upper Limb Functions in Neurological Disorders. J. Neuroeng. Rehabil..

[52] Russo M., Scano A., Brambilla C., d’Avella A. (2024). SynergyAnalyzer: A Matlab Toolbox Implementing Mixed-Matrix Factorization to Identify Kinematic-Muscular Synergies. Comput. Methods Programs Biomed..

[53] Santuz A. (2022). musclesyneRgies: Factorization of Electromyographic Data in R with Sensible Defaults. J. Open Source Softw..

[54] Lapresa M., Zollo L., Cordella F. (2022). A User-Friendly Automatic Toolbox for Hand Kinematic Analysis, Clinical Assessment and Postural Synergies Extraction. Front. Bioeng. Biotechnol..

[55] Zhvansky D.S., Sylos-Labini F., Dewolf A., Cappellini G., d’Avella A., Lacquaniti F., Ivanenko Y. (2022). Evaluation of Spatiotemporal Patterns of the Spinal Muscle Coordination Output during Walking in the Exoskeleton. Sensors.

[56] Rimini D., Agostini V., Knaflitz M. (2017). Intra-Subject Consistency during Locomotion: Similarity in Shared and Subject-Specific Muscle Synergies. Front. Hum. Neurosci..

[57] De Marchis C., Schmid M., Bibbo D., Bernabucci I., Conforto S. (2013). Inter-Individual Variability of Forces and Modular Muscle Coordination in Cycling: A Study on Untrained Subjects. Hum. Mov. Sci..

[58] Zhao K., Zhang Z., Wen H., Scano A. (2021). Intra-Subject and Inter-Subject Movement Variability Quantified with Muscle Synergies in Upper-Limb Reaching Movements. Biomimetics.

[59] Shuman B., Goudriaan M., Bar-On L., Schwartz M.H., Desloovere K., Steele K.M. (2016). Repeatability of Muscle Synergies within and between Days for Typically Developing Children and Children with Cerebral Palsy. Gait Posture.

[60] Maceira-Elvira P., Popa T., Schmid A.-C., Hummel F.C. (2019). Wearable Technology in Stroke Rehabilitation: Towards Improved Diagnosis and Treatment of Upper-Limb Motor Impairment. J. Neuroeng. Rehabil..

[61] Lang C.E., Barth J., Holleran C.L., Konrad J.D., Bland M.D. (2020). Implementation of Wearable Sensing Technology for Movement: Pushing Forward into the Routine Physical Rehabilitation Care Field. Sensors.

[62] Bruton M., O’Dwyer N. (2018). Synergies in Coordination: A Comprehensive Overview of Neural, Computational, and Behavioral Approaches. J. Neurophysiol..

[63] Cheung V.C.K., Seki K. (2021). Approaches to Revealing the Neural Basis of Muscle Synergies: A Review and a Critique. J. Neurophysiol..

[64] Singh R.E., Iqbal K., White G., Hutchinson T.E. (2018). A Systematic Review on Muscle Synergies: From Building Blocks of Motor Behavior to a Neurorehabilitation Tool. Appl. Bionics. Biomech..

[65] Turpin N.A., Uriac S., Dalleau G. (2021). How to Improve the Muscle Synergy Analysis Methodology?. Eur. J. Appl. Physiol..

[66] Scano A., Mira R.M., d’Avella A. (2022). Mixed Matrix Factorization: A Novel Algorithm for the Extraction of Kinematic-Muscular Synergies. J. Neurophysiol..

[67] O’Reilly D., Delis I. (2024). Dissecting Muscle Synergies in the Task Space. eLife.

[68] Lapresa M., Corradini V., Iacca A., Scotto di Luzio F., Zollo L., Cordella F. (2024). A Comprehensive Analysis of Task-Specific Hand Kinematic, Muscle and Force Synergies. Biocybern. Biomed. Eng..

[69] Scano A., Dardari L., Molteni F., Giberti H., Tosatti L.M., d’Avella A. (2019). A Comprehensive Spatial Mapping of Muscle Synergies in Highly Variable Upper-Limb Movements of Healthy Subjects. Front. Physiol..

[70] Irastorza-Landa N., García-Cossio E., Sarasola-Sanz A., Brötz D., Birbaumer N., Ramos-Murguialday A. (2021). Functional Synergy Recruitment Index as a Reliable Biomarker of Motor Function and Recovery in Chronic Stroke Patients. J. Neural. Eng..

[71] Funato T., Hattori N., Yozu A., An Q., Oya T., Shirafuji S., Jino A., Miura K., Martino G., Berger D. (2022). Muscle Synergy Analysis Yields an Efficient and Physiologically Relevant Method of Assessing Stroke. Brain Commun..

[72] Camardella C., Barsotti M., Buongiorno D., Frisoli A., Bevilacqua V. (2021). Towards Online Myoelectric Control Based on Muscle Synergies-to-Force Mapping for Robotic Applications. Neurocomputing.

